# On the analysis of phylogenetically paired designs

**DOI:** 10.1002/ece3.1406

**Published:** 2015-01-30

**Authors:** Jennifer L Funk, Cyril S Rakovski, J Michael Macpherson

**Affiliations:** Schmid College of Science and Technology, Chapman UniversityOrange, California, 92866

**Keywords:** ANOVA, mixed model, parameter estimation, phylogenetically controlled designs

## Abstract

As phylogenetically controlled experimental designs become increasingly common in ecology, the need arises for a standardized statistical treatment of these datasets. Phylogenetically paired designs circumvent the need for resolved phylogenies and have been used to compare species groups, particularly in the areas of invasion biology and adaptation. Despite the widespread use of this approach, the statistical analysis of paired designs has not been critically evaluated. We propose a mixed model approach that includes random effects for pair and species. These random effects introduce a “two-layer” compound symmetry variance structure that captures both the correlations between observations on related species within a pair as well as the correlations between the repeated measurements within species. We conducted a simulation study to assess the effect of model misspecification on Type I and II error rates. We also provide an illustrative example with data containing taxonomically similar species and several outcome variables of interest. We found that a mixed model with species and pair as random effects performed better in these phylogenetically explicit simulations than two commonly used reference models (no or single random effect) by optimizing Type I error rates and power. The proposed mixed model produces acceptable Type I and II error rates despite the absence of a phylogenetic tree. This design can be generalized to a variety of datasets to analyze repeated measurements in clusters of related subjects/species.

## Introduction

In the last decade, the number of phylogenetically controlled experimental designs and statistical analyses has increased dramatically in the field of ecology (e.g., Agrawal et al. 2005; Funk and Vitousek 2007; Heard and Sax 2013). Controlling for phylogenetic relatedness among a suite of species is important because similarities in traits or responses among species may result from biological and ecological factors or may be strongly affected by shared evolutionary history. Statistically speaking, we cannot assume that species are independent samples drawn from the same distribution if many species share common genetic ancestry (Felsenstein 1985). When conventional statistics are applied to comparative data, the overestimate of independent observations leads to inflated Type I error rates (i.e., statistical significance claimed too often; Garland et al. 2005).

Adding phylogenetic information into analyses or experimental designs is essential to tease apart the influence of ecological and evolutionary factors on our trait or response of interest. For example, the leaf economics spectrum identifies strong correlations among leaf metabolic processes and structure across a broad taxonomic range of species resulting from biophysical constraints on leaves (Reich et al. 1997). Using a phylogenetically controlled analysis, Ackerly and Reich (1999) found that, overall, these correlations were not an artifact of shared evolutionary history at either end of the leaf economics spectrum (e.g., thick-leaved species with low photosynthetic rates occur in closely related genera). However, correlations among a few leaf-level traits were reduced when phylogenetic information was considered. For example, the correlation between leaf life span and leaf area was driven by large differences in these traits between angiosperms and conifers – there was very little variation in these traits within these plant groups (Ackerly and Reich 1999).

A number of phylogenetically based statistical approaches derived from standard regression techniques are commonly employed for comparative biological analysis (Garland et al. 2005; Rezende and Diniz-Filho 2012). For example, phylogenetic independent contrasts (PIC) calculate the estimated differences in traits between sister taxa descended from each node of a phylogeny and then evaluate trait correlations between these contrasts (Felsenstein 1985). A critical component of these approaches is a resolved phylogeny and, in particular, relative branch lengths (the length of time since two species shared a common ancestor; Felsenstein 1985), although some simulations have shown that alternative phylogenies, including ones where random resolutions are used to construct uncertain portions of phylogenies, may have little influence on the outcome of PIC (Donoghue and Ackerly 1996; Ackerly and Reich 1999). Conversely, Davies et al. (2012) recently found that incompletely resolved phylogenies are more likely to inflate estimates of phylogenetic conservatism. However, creating resolved phylogenies is usually beyond the scope of many ecological studies.

At the expense of statistical power, phylogenetically paired designs circumvent the need for resolved phylogenies (Ackerly 2000; Maddison 2000). Phylogenetically paired designs invoke paired contrasts of closely related species, most often congeners (Table1), and have proved valuable in understanding a range of ecological issues (e.g., Garnier 1992; Agrawal et al. 2005; Fine et al. 2006; Bhaskar et al. 2007; Funk and Vitousek 2007). Differences between the species in the pairs are phylogenetically independent, just like PIC, but there is no information about the time since their most recent common ancestor. While the two species within a pair need to be more closely related to each other than to any other species in the sample, contrasts would not necessarily have equal variances (Felsenstein 1985).

**Table 1:** An example of a phylogenetically paired design, where pairs of closely related species (within genera or family) are compared. In this example, ecologically equivalent, closely related native and invasive species in Hawaii are compared (Funk and Throop 2010)

Family	Invasive species	Native species
Asteraceae	*Conyza canadensis*	*Pseudognaphalium sandwicensium*
	Ageratina riparia	*Dubautia scabra*
	*Hypochoeris radicata*	*Argyroxiphium kauense*
Fabaceae	Desmodium sandwicense	*Sesbania tomentosa*
	*Leucaena leucocephala*	*Sophora chrysophylla*
	*Prosopis pallida*	*Erythrina sandwicensis*
Myrtaceae	Psidium cattleianum	*Metrosideros polymorpha*
Nephrolepidaceae	Nephrolepsis multiflora	*Nephrolepsis cordifolia*
Oleaceae	Olea europaea	*Nestegis sandwicensis*
Plantaginaceae	Plantago lanceolata	*Plantago hawaiiensis*
Poaceae	*Rhynchelytrum repens*	*Heteropogon contortus*
	*Ehrharta stipoides*	*Isachne distichophylla*
	Holcus lanatus	*Deschampsia nubigena*
	*Paspalum urvillei*	*Eragrostis variabilis*
Rosaceae	*Pyracantha angustifolia*	*Osteomeles anthyllidifolia*
	*Rubus ellipticus*	*Rubus hawaiiensis*
Solanaceae	*Nicotiana glauca*	*Nothocestrum brevifolia*
Within-order comparisons		
Sapindales	*Schinus terebinthifolius (Anacardiaceae)*	*Dodonaea viscosa (Sapindaceae)*
Asparagales	*Crocosmia pottsii x aurea (Iridaceae)*	*Astelia menziesiana (Asteliaceae)*

Phylogenetically paired designs have been used to address a diverse set of ecological questions, including differences between the annual and perennial life form (Garnier 1992) and adaptation to environmental factors (Fine et al. 2006; Bhaskar et al. 2007). The paired design is now especially widespread in studies of invasive species, where introduction history may have resulted in different phylogenetic makeup between native and invasive species groups. In order to test hypotheses pertaining to differences between native and invasive species (e.g., growth rates, susceptibility to insect damage), researchers must control for phylogenetic differences between these groups of species. For example, to identify traits that permit invasiveness, researchers have used phylogenetic comparative designs to minimize trait differences associated with comparing unrelated species and disparate life forms (Burns and Winn 2006; Muth and Pigliucci 2006; Richards et al. 2006; Funk and Vitousek 2007; Grotkopp and Rejmanek 2007; Funk 2008). In addition, controlling for phylogenetic relatedness provides a conservative test of enemy impact on invasions because alien species that are related to native species are more likely to acquire enemies present on their native relatives (Agrawal et al. 2005; Parker and Gilbert 2007; Engelkes et al. 2008; Dawson et al. 2009).

The analysis of the phylogenetically paired design has been nearly as diverse as its application. Analyses include paired t-tests (Hoffmann et al. 2003; Burns and Winn 2006), nested designs with effects nested within the phylogenetic term (Burns 2006; Funk and Vitousek 2007), mixed model ANOVA with pair as a random effect (Fine et al. 2006; Bhaskar et al. 2007; ten Brink et al. 2013), one-, two-, or three-factor ANOVA with pair as a fixed effect (Blaney and Kotanen 2001; Agrawal et al. 2005; Fine et al. 2006; Engelkes and Mills 2013), designs that do not include pair in the model (Garnier 1992; Funk 2008; Heard and Sax 2013) and many others (Kempel et al. 2013; Kirichenko et al. 2013). In this study, we propose a mixed model approach for the analysis of such phylogenetically paired data. The inclusion of two random effects for pair and species within pair introduce a “two-layer” compound symmetry variance structure (explained below) that addresses both the correlations within the pairs of related species and the correlations between the pairs of repeated measurements within species.

Our goal is not to evaluate the paired contrast method itself, but to critically evaluate the analysis of this design. Because paired contrasts will differ in their relatedness and potentially have unequal variance (Felsenstein 1985), phylogenetically based statistical approaches should be the preferred method of analysis when resolved phylogenies are available (Garland et al. 2005). However, in the common case that reliable resolved phylogenies are not available, the type of analysis we propose is an acceptable way to add relatedness into the interpretation of ecological datasets.

## Materials and Methods

### The sample dataset

We analyzed four leaf traits pertaining to herbivore defense among pairs of native and invasive plant species: toughness, thickness, nitrogen content, and phenolic content (for details on trait collection, see Funk and Throop 2010). Briefly, we selected 19 pairs of phylogenetically related native and invasive species occurring in three habitat types on the Island of Hawaii. In total, there were three congeneric, fourteen confamilial, and two within-order comparisons (Table1). Confamilial pairs may not be each other's closest relatives within family but were compared because they had similar growth form and co-occurred at the same site (similar light, precipitation, elevation, and soil substrate age). We selected five individuals per species which provided a good representation of plants within a site. We included varying levels of relatedness within pairs (e.g., genus versus family level) in order to maximize the number of co-occurring related native and invasive species pairs.

### The model

We propose the use of a mixed model approach for the analysis of replicated pairwise-dependent experimental units. For instance, in the provided illustrative dataset (Table1), the pairs consist of taxonomically similar species and several outcome variables of interest were measured on five plants per species. Pair must be included as a random effect to account for the correlations between all measurements within a pair which are induced by their common genetic ancestry. Similarly, species must be included as a random effect to account for the correlations between all pairs of repeated measurements on the same species. Thus, the presence of these two random effects introduces an appropriate covariance structure on the data, a two-layer compound symmetry that reflects the correlations between observations between species within pair and repeated measurements within species (Laird and Ware 1982). In other words, this structure implies that all replicates from within a species are equally correlated with each other, and that the total variation can be partitioned into a shared within-species component and an unshared component. An advantage of a compound symmetry structure is that only two variance parameters need to be estimated. Covariance structures that lack compound symmetry estimate many parameters and yield less power; however, they are valuable in datasets where, for example, correlations among repeated measures decay over time in a nonparametric fashion. The compound symmetry structure would not be appropriate if there were a time component to the measurements; that is, if the same plants were sampled at different points in time. The study design analyzed here does not have a time component.

More formally, let *y*_*ijk*_ denote the *k*-th measurement (replicate plant) of the *j*-th species from the *i*-th pair. “Measurement” refers to any dependent variable of interest, such as the leaf traits that will be considered in our sample dataset below. Then, the proposed model is given by, *y*_*ijk*_ = *μ* + *βx*_*ij*_ + *α*_*i*_ + *γ*_*ij*_ + *ε*_*ijk*_, where *μ* is the intercept coefficient, *β* is the coefficient for the fixed effect of origin (native or invasive), *x*_*ij*_ is an indicator variable representing species origin, and *α*_*i*_ ∽ *N*(0, *τ*^2^), *γ*_*ij*_ ∽ *N*(0, *ω*^2^), *ε*_*ijk*_ ∽ *N*(0, *σ*^2^) are independent random effects for pair, species and error, respectively.

In essence, this model defines a block-diagonal covariance structure where each block is parsimoniously parameterized to represent the covariance among all observations and within pairs such that all correlations within a species are equal to *ρ* = (*τ*^2^ + *ω*^2^)/(*τ*^2^ + *ω*^2^ + *σ*^2^) and all correlations between species within a pair are equal to *θ* = *τ*^2^/(*τ*^2^ + *ω*^2^ + *σ*^2^). Thus, the three random effects (pair, species, and observation within species) induce the following structure on the covariance matrix ∑,

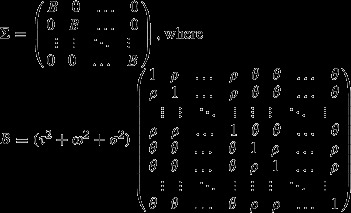


As is standard with mixed models, we estimated model parameters using restricted maximum likelihood (REML) with the lme4 package. Complex variance–covariance structure and small sample sizes may cause mixed model *F*-statistics to fail to follow an *F*-distribution, which can lead to erroneous inferences (Kenward and Roger 1997). Thus, we assessed significance using the method of Kenward and Roger (1997). We note that this choice was appropriate as we have considered balanced designs here; were the design unbalanced, or if there were many missing data, a parametric bootstrap approach to assessing significance would be more appropriate (Davison and Hinkley 1997).

We analyzed our sample dataset of four leaf traits from 19 pairs of phylogenetically related native and invasive species with a mixed model with a fixed effect of origin (native or invasive) and two random effects (pair, species) to derive realistic simulation parameters (e.g. see Simulation section below). Parameter values for each model were evaluated using REML and significance testing approaches described above. The code for all R analyses is presented as Supporting Information.

### Simulation

We performed a simulation study to assess the effect of model misspecification on the standard error of the regression coefficients and the corresponding effects on the Type I and II error rates. The variance estimates present the essential issue with such complex data as the analyzed model misspecifications yield unbiased estimates of the regression coefficients. We simulated 1000 datasets for each combination of variance components and analyzed them via three competing model strategies: (1) a fixed effect one-way ANOVA, (2) a mixed effects model with random effect for pair, and (3) a mixed effects model with random effects for species and pair. We propose that model 3 is the most appropriate for our experimental design. Models 1 and 2 suffer from pseudo-replication (if replicates within species are included in the analysis). Additionally, Model 2 underestimates the between- species within pair correlation, which one might call “pseudo-independence”. As described above, these and other models have been and continue to be used in the ecological literature (e.g., Agrawal et al. 2005; Heard and Sax 2013).

We simulated trait values for evolutionarily related species pairs from the proposed mixed model. Namely, trait value was simulated as the sum of four terms: a fixed effect due to origin, a random effect due to pair, a random effect due to species, and an error term. The effect of origin was prespecified, the random effect due to species was one draw from the normal distribution with prespecified variance per species, and the error term was one draw from the normal distribution with prespecified variance per replicate. The random effect due to pair is where phylogenetic relatedness enters the simulation. One phylogeny was simulated under the Yule model using the apTreeshape package per run, having as many tips as the specified number of species pairs. Brownian trait evolution on this tree was then simulated using the ape package. The trait values were scaled such that the variance within pairs was equal to the *α* specified.

**Figure 1:**
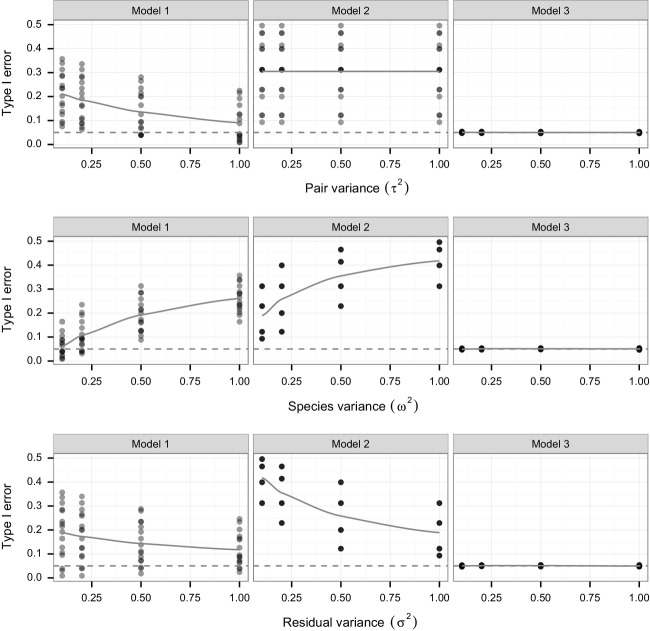
Simulated results of different variance components (*τ*^2^, *ω*^2^, *σ*^2^) on Type I error rates for the three models. Variance components were set, respectively, to 0.1, 0.2, 0.5, and 1.0 and results include all combinations (*n* = 64 for each panel). The models applied to all simulated datasets are: (1) a fixed effect one-way ANOVA, (2) a mixed effects model with random effect for pair, and (3) a mixed effects model with random effects for species and pair. The solid gray line is a smoothed estimate of Type 1 error. The dashed gray line is set at *α* = 0.05, the nominal significance threshold.

In the simulation results shown in Figs.1 and S1, the number of pairs and the number of repeated measurements in the datasets were fixed at 30 and 5, respectively. For simulations under the alternative, the effect of invasive versus native species (*β*) was fixed at 0.5 (Fig. S1). We show results for each combination of four random effect sizes across the three random effects, motivated by the values present in the Funk and Throop (2010) dataset: *τ*^2^, *ω*^2^, and *σ*^2^ were each set to 0.1, 0.2, 0.5, and 1.0.

## Results

We evaluated the performance of three models (two reference models that are commonly used to analyze phylogenetically paired designs and our proposed model) using simulated data that explicitly incorporated a phylogenetic tree to represent correlations between species and pair that mimic those found in nature. We found that the two reference models, Model 1 and Model 2, had inflated Type I error rates (Fig.1). The Type I error of Model 2 (maximum 40%) was typically higher than that of Model 1 (maximum 25%). As pair variance increased, Type I error decreased for Model 1, but was unaffected in Model 2. As species variance increased, Type I error increased in both Model 1 and 2. As residual variance increased, the Type I error of both Model 1 and 2 decreased. By contrast, Model 3 was unaffected by variation in any of the three variance components and its Type I error remained close to the alpha of 0.05 (Fig.1).

Figure S1 shows the power of the three models under the simulation for a fixed effect size of origin at 0.5. As expected, the power declined as the three variance components increased. While Models 1 and 2 had higher power than Model 3 under some scenarios (e.g., high species and residual variance, Fig. S1), this can be disregarded in light of the severe Type I error rate inflation.

The sample dataset from Funk and Throop (2010) was also analyzed using each of the three models (Table2). The effect due to origin would be regarded as significant under all models for this particular dataset. However, consistent with the observed Type I error (Fig.1), the *P*-values assessed under Models 1 and 2 are markedly smaller than those of Model 3.

**Table 2:** The analysis of Funk and Throop's (2010) dataset using Model 1 (origin as a fixed effect), Model 2 (origin as a fixed effect and pair as a random effect), and Model 3 (origin as a fixed effect, pair and species as random effects). Restricted maximum likelihood (REML) was used to estimate parameter values for the mixed models, Model 2 and Model 3 (estimate for the fixed origin effect is shown). For clarity, random effects are not shown. The significance values for the mixed models were estimated using the method of Kenward and Roger (1997)

	Effect of Origin		
	Model 1	Model 2	Model 3
Leaf toughness	72.66***	72.04***	66.76*
Leaf thickness	0.004***	0.004***	0.004*
Leaf nitrogen content	−0.514***	−0.493***	−0.491**
Leaf phenolic content	−1.995**	−2.019***	−1.997*

* *P* < 0.05

** *P* < 0.01

*** *P* < 0.001.

## Discussion

Phylogenetically paired designs circumvent the need for resolved phylogenies in phylogenetically based regression techniques; however, the analysis of paired designs has not been critically evaluated and ecologists have used many different approaches (e.g., Burns and Winn 2006; Fine et al. 2006; Funk and Vitousek 2007; Heard and Sax 2013; Kempel et al. 2013). In this study, we performed a biologically motivated simulation to evaluate two commonly used models alongside our proposed model, which treated species and pair as random effects and introduced a compound symmetry variance structure that addressed the correlations of the data within these categories resulting from genetic relatedness. We found that our proposed model had much better operating characteristics and was less prone to false positives (i.e., Type I errors).

Our two reference models (Models 1 and 2) have been used to analyze ecological datasets (e.g., Fine et al. 2006; Bhaskar et al. 2007) but possess disadvantageous characteristics associated with increased Type I error. Because Model 1 assumes no correlations within species or between species within pair, it underestimates these correlations, resulting in high Type I error. Model 2's covariance structure forces a single parameter to represent both within species and between species within pair correlations; thus, Model 2 will likely underestimate the within species correlation, like Model 1, but in contrast will overestimate the between species within pair correlation. The underestimation and overestimation in Model 2 combine in a complex way to produce an inflated test statistic, resulting in greater Type I error. While outside the scope of this study, it may be possible to analytically confirm the patterns we observed in our simulation by expressing the test statistics as functions of the vector of observations, design matrix, and correlation parameters that appear in the covariance structure.

In contrast, the proposed model with two-layer compound symmetry variance structure (Model 3) attains ideal Type I error rates in all scenarios we examined. Additionally, this model preserves the phylogenetic structure of the data and permits use of all replicates. Accounting for correlations among species within pair will be particularly important as variation among species increases, as evidenced by high Type I error in Models 1 and 2 under conditions of high species variance (Fig.1). That said, a random effect for species may not need to be incorporated if studies summarize data at the species level (i.e., if repeated measures made on multiple individuals are averaged within each species).

When analyzing ecological datasets that include large numbers of species, it may be difficult to obtain a detailed phylogeny. An interesting result from this study is that Model 3 had good Type I and II errors, despite the absence of a phylogenetic tree. Model 3 adds just a single parameter to the covariance matrix to capture all phylogenetic relatedness, but the model does an adequate job, at least as far as these simulations are concerned. Comparing the performance of Model 3 with methods that use resolved phylogenies is an opportunity for future research.

In conclusion, the choice of linear model has a pronounced effect on the inference of phylogenetically paired data. Using a mixed model with pair and species as random effects leads to an appropriate variance structure. While the linear mixed effect model is a well-known method for data analysis in the ecological literature, the appropriate statistical treatment of a paired design is not obvious and does not appear in any of the common ecological statistics books (e.g., Quinn and Keough 2002). The method proposed here can be generalized to a variety of datasets to analyze repeated measurements in clusters of related subjects/species.

## Conflict of Interest

None declared.

## Supporting Information

**Figure S1.** Simulated results of different variance components (τ_2_, ω_2_, σ_2_) on power for the three models. **Appendix S1.** R script that simulates phylogenetically-paired data. **Appendix S2.** Shell script to run evaluate_models.R over a grid of parameters. **Appendix S3.** R script that evaluates Model 1, Model 2, and Model 3 for each of four traits from Funk and Throop (2010). **Table S1.** Dataset from Funk and Throop (2010).
