# Supplementary material for: On the analysis of phylogenetically paired designs
Source: Ecol Evol. 2015 Jan 30;5(4):940–7. doi: 10.1002/ece3.1406 (PMC4338975; doi:10.1002/ece3.1406)
Supplement: Supplementary file 1 [file ece30005-0940-sd1.pdf]

Supporting Information
Table of contents

Figure S1: Simulated results of different variance components on power for the three models.

Appendix S1: R script that simulates phylogenetically-paired data.

Appendix S2: Shell script to run evaluate_models.R over a grid of parameters.

Appendix S3: R script that evaluates Model 1, Model 2 and Model 3 for each of four traits from Funk and Throop (2010).

Table S1: Dataset from Funk and Throop (2010).

Note: Supporting Information is also available at Github.
https://github.com/mmacpherson/funk-etal-mixed-model-study

Figure S1. Simulated results of different variance components (τ^2, ω^2, σ^2) on power for the three models. Variance components were set respectively to 0.1, 0.2, 0.5 and 1.0 and results include all combinations (n=64 for each panel). The fixed effect of origin was set at 0.5 throughout. The models applied to all simulated datasets are: (1) a fixed effect one-way ANOVA, (2) a mixed effects model with random effect for pair, and (3) a mixed effects model with random effects for species and pair. The solid gray line is a smoothed estimate of power.

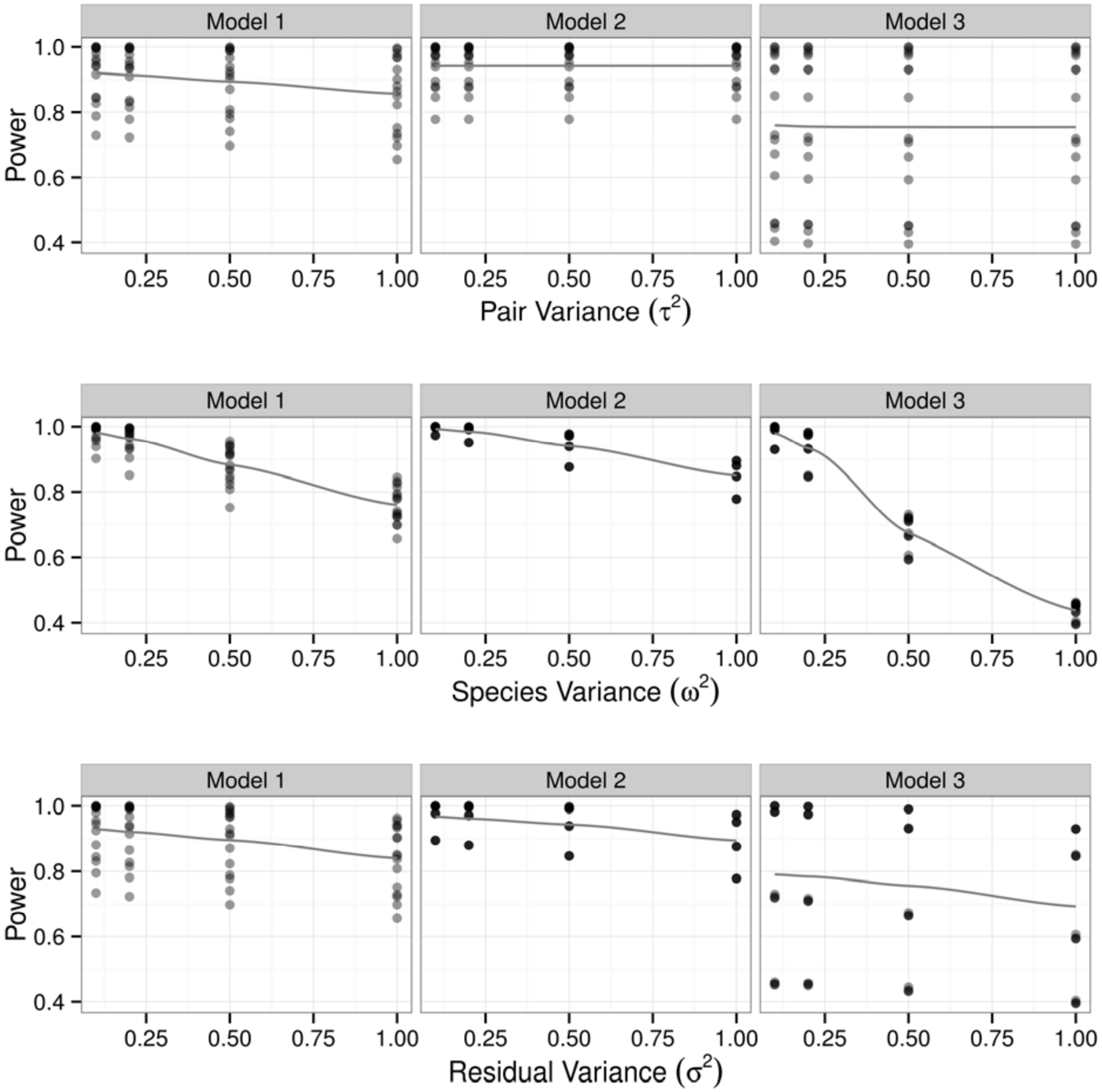

Appendix S1 - evaluate_models.R
Description: R script that simulates phylogenetically-paired data. Fits and assesses for significance Model 1, Model 2, and Model 3 from the paper.

```
library(ape)          # -- provides rcoal, rtree
library(apTreeshape)  # -- provides rtreeshape
library(geiger)       # -- provides sim.char
library(lme4)         # -- provides lmer
library(afex)         # -- provides mixed, for p-values

## -- ensure reproducibility
set.seed(2009)

## -- process CLI args
ca <- commandArgs()[-(1:5)]
ca <- as.numeric(ca)

pair.var     <- ca[1]
species.var  <- ca[2]
residual.var <- ca[3]
effect.size  <- ca[4]
nsim         <- as.integer(ca[5])

pval.method <- "KR" # -- use Kenward-Roger for mixed models

## -- simulation parameters
npairs      <- 30
nspecies    <- 2 * npairs
nreplicates <- 5

pair.sd     <- sqrt(pair.var)
pair.sd     <- 1.0
species.sd  <- sqrt(species.var)
residual.sd <- sqrt(residual.var)

## -- origin offset vector
vorigin <- c(0.0, effect.size)

## -- Extract same parameters from lmer model as summary()$coefs does from lm
lmer.summary <- function(fitted) {
    fitted.est <- fixef(fitted)[2]
    fitted.sd  <- sqrt(diag(vcov(fitted)))[2]
    fitted.t   <- fitted.est / fitted.sd
    fitted.p   <- 2 * pnorm(-abs(fitted.t))
    c(fitted.est, fitted.sd, fitted.t, fitted.p)
}

## -- Output matrix containing estimates, variances, tests and p-values for
the three methods
outmat <- matrix(0, nsim, 12)

for (k in 1:nsim)
{
    ## -- generate Yule phylogenetic tree
    tree <- as.phylo(rtreeshape(1, npairs, model="yule")[[1]])
```

```
    ## -- pair offset vector, via evolutionary tree simulation
    vpair <- sim.char(tree, matrix(c(pair.sd ** 2)))[1:npairs,1,1]
    vpair <- vpair * sqrt(pair.var) / sd(vpair)

    ## -- species offset vector
    vspecies <- rnorm(nspecies, sd=species.sd)

    ## -- allocate vectors to hold the simulated data
    value     <- c()
    origin    <- c()
    pair      <- c()
    replicate <- c()
    species   <- c()

    ## -- simulate dataset!
    for (.origin in 1:2) {
        for (.pair in 1:npairs) {
            for (.replicate in 1:nreplicates) {
                species.index <- 1 + 2 * (.pair - 1) + (.origin - 1)
                components <- c(vorigin[.origin],        # -- fixed effect of
origin
                                vpair[.pair],            # -- random effect
of pair
                                vspecies[species.index], # -- random effect
of species
                                rnorm(1, sd=residual.sd) # -- residual
(random) effect
                                )

                ## -- augment vectors
                value     <- c(value, sum(components))
                origin    <- c(origin, .origin)
                pair      <- c(pair, .pair)
                replicate <- c(replicate, .replicate)
                species   <- c(species, species.index)
            }
        }
    }

    origin <- factor(origin) # -- or else afex::mixed will complain

    model1 <- lm(value ~ origin)
    data <- data.frame(value=value, origin=origin, pair=pair,
species=species)
    model2 <- mixed(value ~ origin + (1|pair),
                    data,
                    check.contrasts=FALSE,
                    method=pval.method, progress=FALSE)
    model3 <- mixed(value ~ origin + (1|pair) + (1|species),
                    data,
                    check.contrasts=FALSE,
                    method=pval.method, progress=FALSE)

    outmat[k,1:4]  <- summary(model1)$coef[2,]
    outmat[k,5:8]  <- c(0,0,0,model2$anova.table$p.value)
    outmat[k,9:12] <- c(0,0,0,model3$anova.table$p.value)
```

```
}

# -- Compute Type I error when effect size is zero, power when it's not
m1 <- sum(outmat[,4] < 0.05) / nsim
m2 <- sum(outmat[,8] < 0.05) / nsim
m3 <- sum(outmat[,12] < 0.05) / nsim

# -- Summarize
c(pair.var, species.var, residual.var, effect.size, m1, m2, m3)
```

Appendix S2
Description: Shell script to run evaluate_models.R over a grid of parameters.

```
#!/bin/sh

TR=table3_raw.txt
rm -f $TR

NSIM=1000

for PAIRVAR in 0.1 0.2 0.5 1.0
do
    for SPECIESVAR in 0.1 0.2 0.5 1.0
    do
        for RESIDUALVAR in 0.1 0.2 0.5 1.0
        do
            for EFFECTSIZE in 0.0 0.5
            do
                Rscript evaluate_models.R $PAIRVAR $SPECIESVAR $RESIDUALVAR
$EFFECTSIZE $NSIM 2> /dev/null | cut -f2- -d " " >> $TR
            done
        done
    done
done
```

Appendix S3

Description: Evaluates Model 1, Model 2, and Model 3 for each of four traits from Funk & Throop (2010).

```
## -- Setup.
library(afex) # -- provides mixed, wrapper around lme4
pval.method <- "KR" # - ie, Kenward-Roger


herb <- read.table("Funk_2010_herbivory_study.csv", h=T, sep=",")

traits <- c("phenolics", "toughness", "thickness", "leafN")
results <- c()
for (trait in traits) {

    herb["trait"] <- herb[trait]

    model1.trait <- lm(trait ~ origin, herb)
    model2.trait <- mixed(trait ~ origin + (1|pair),
                          herb,
                          check.contrasts=FALSE,
                          method=pval.method,
                          progress=FALSE)
    model3.trait <- mixed(trait ~ origin + (1|pair) + (1|species),
                          herb,
                          check.contrasts=FALSE,
                          method=pval.method,
                          progress=FALSE)

    vals <- c(

        ## -- pull out fixed effect due to origin
        as.numeric(model1.trait$coefficients[2]),
        model2.trait$full.model@beta[2],
        model3.trait$full.model@beta[2],

        ## -- pull out p.value
        summary(model1.trait)$coef[2,4],
        model2.trait$anova.table$p.value,
        model3.trait$anova.table$p.value

    )
    ## -- append results
    results <- rbind(results, t(matrix(vals, nrow=2, byrow=T)))
}

## -- rearrange into coherent data frame & write to disk
results <- as.data.frame(results)
names(results) <- c("origin", "p.value")

results$trait <- rep(traits, each=3)
results$model <- rep(c("model.one", "model.two", "model.three"), 4)

results <- results[,c(3,4,1,2)]
write.table(results,
"funk_traits_model_fits.csv",quote=F,sep=",",row.names=F)
```

Table S1

Description: Dataset from Funk & Throop (2010).

pair	species	origin	replicate	phenolics	toughness	thickness	leafN
1	agri	invasive	1	16.11085	88.33333	0.00775	1.160433
1	agri	invasive	2	14.96297	106.6667	0.00825	1.345337
1	agri	invasive	3	13.93591	116.6667	0.006417	1.148372
1	agri	invasive	4		133.3333	0.007583	1.276154
1	agri	invasive	5		90	0.0075	
1	dusc	native	1	10.43184	421.6667	0.012333	1.125327
1	dusc	native	2	9.465202	380	0.010167	0.86125
1	dusc	native	3	10.49226	408.3333	0.021417	1.071
1	dusc	native	4		340	0.01675	1.322222
1	dusc	native	5		400	0.01725	0.898333
2	coca	invasive	1	6.806942	40	0.004583	1.353087
2	coca	invasive	2	8.438147	20	0.006167	1.447405
2	coca	invasive	3	7.109017	15	0.0065	1.281424
2	coca	invasive	4		11.66667	0.005167	1.730788
2	coca	invasive	5		41.66667	0.005417	1.522471
2	pssa	native	1	5.538227	70	0.004083	0.806435
2	pssa	native	2	7.531922	96.66667	0.005583	0.798469
2	pssa	native	3	8.377732	106.6667	0.00725	1.240652
2	pssa	native	4		83.33333	0.00525	1.14795
2	pssa	native	5		51.66667	0.007333	0.841964
3	asme	native	1	2.940382	411.6667	0.018125	1.199378
3	asme	native	2	2.940382	430	0.01575	0.882756
3	asme	native	3	2.577892	430	0.01975	1.064676
3	asme	native	4		373.3333	0.01625	1.038543
3	asme	native	5		428.3333	0.016125	
3	crpo	invasive	1	6.625697	78.33333	0.006	0.726259
3	crpo	invasive	2	5.900717	140	0.00675	0.848439
3	crpo	invasive	3	7.350677	183.3333	0.00675	1.083104
3	crpo	invasive	4		85	0.0045	1.098558
3	crpo	invasive	5		116.6667	0.007	
4	desa	invasive	1	15.86919	146.6667	0.013667	1.535742
4	desa	invasive	2	12.30471	188.3333	0.008111	2.231068
4	desa	invasive	3	11.4589	153.3333	0.012111	1.623666
4	desa	invasive	4		96.66667	0.012333	2.191972
4	desa	invasive	5		88.33333	0.012444	
4	seto	native	1	4.813247	156.6667	0.015444	2.168535
4	seto	native	2	4.148682	96.66667	0.014556	2.298273

4	seto	native	3	4.571587	123.3333	0.013222	1.705634
4	seto	native	4		88.33333	0.013889	2.175165
4	seto	native	5		101.6667	0.015444	2.24842
5	ehst	invasive	1	2.457062	105	0.003167	0.615303
5	ehst	invasive	2	2.396647	161.6667	0.003083	0.859784
5	ehst	invasive	3	2.457062	213.3333	0.0035	0.633253
5	ehst	invasive	4		146.6667	0.004667	0.514336
5	ehst	invasive	5		138.3333	0.005417	0.718496
5	isdi	native	1	3.242457	300	0.006875	0.688302
5	isdi	native	2	3.182042	250	0.00725	0.684521
5	isdi	native	3	3.242457	311.6667	0.00725	0.651839
5	isdi	native	4		290	0.007833	0.582426
5	isdi	native	5		270	0.007917	0.776702
6	denu	native	1	2.034157	430	0.016917	2.054794
6	denu	native	2	2.275817	430	0.020417	1.885813
6	denu	native	3	2.154987	430	0.01825	2.504821
6	denu	native	4		430	0.017667	3.017015
6	denu	native	5		430	0.01875	1.64475
6	hola	invasive	1	4.390342	146.6667	0.007833	1.014364
6	hola	invasive	2	5.115322	111.6667	0.00825	1.16659
6	hola	invasive	3	4.692417	80	0.008333	1.183109
6	hola	invasive	4		63.33333	0.00825	1.29375
6	hola	invasive	5		70	0.006417	0.806745
7	arka	native	1	5.961132	430	0.038875	1.786667
7	arka	native	2		402.5	0.045125	1.593239
7	arka	native	3		402.5	0.05275	1.871477
7	arka	native	4		402.5	0.049875	1.381333
7	arka	native	5		410	0.040375	
7	hyra	invasive	1	7.048602	55	0.010833	0.954626
7	hyra	invasive	2	6.686112	46.66667	0.009167	0.925431
7	hyra	invasive	3	6.021547	106.6667	0.010167	0.824616
7	hyra	invasive	4		68.33333	0.01	1.412523
7	hyra	invasive	5		98.33333	0.009917	
8	lele	invasive	1	9.042297	16.66667	0.009333	3.521006
8	lele	invasive	2	8.740222	23.33333	0.011167	3.872293
8	lele	invasive	3	6.927772	11.66667	0.010333	2.95441
8	lele	invasive	4		16.66667	0.011167	3.780965
8	lele	invasive	5		5	0.010333	2.927629
8	soch	native	1	3.907022	148.3333	0.014667	1.991473
8	soch	native	2	3.786192	170	0.011	2.204119
8	soch	native	3	3.846607	118.3333	0.012556	2.013125

8	soch	native	4		46.66667	0.013444	1.861032
8	soch	native	5		163.3333	0.012889	
9	neco	native	1	3.302872	46.66667	0.008667	0.831356
9	neco	native	2	3.484117	50	0.01125	0.471465
9	neco	native	3	3.484117	95	0.007917	0.623547
9	neco	native	4		91.66667	0.010083	1.138343
9	neco	native	5		113.3333	0.01225	0.960819
9	nemu	invasive	1	3.665362	53.33333	0.00875	0.73628
9	nemu	invasive	2	2.819552	66.66667	0.01	0.524386
9	nemu	invasive	3	3.302872	128.3333	0.009667	0.937435
9	nemu	invasive	4		195	0.01	0.813559
9	nemu	invasive	5		53.33333	0.008167	0.691342
10	nigl	invasive	1	5.356982	45	0.010778	1.999235
10	nigl	invasive	2	4.027852	56.66667	0.013222	1.401716
10	nigl	invasive	3	5.659057	86.66667	0.011	2.304274
10	nigl	invasive	4		63.33333	0.011222	2.114746
10	nigl	invasive	5		11.66667	0.010444	
10	nobr	native	1	5.900717	55	0.013333	3.872669
10	nobr	native	2	6.081962	50	0.0145	2.541174
10	nobr	native	3		51.66667	0.012333	2.578485
10	nobr	native	4		138.3333	0.008222	1.449467
10	nobr	native	5		78.33333	0.014444	
11	nesa	native	1	5.417397	93.33333	0.010222	2.166162
11	nesa	native	2	4.088267	298.3333	0.012778	2.165501
11	nesa	native	3	4.209097	166.6667	0.013333	2.739696
11	nesa	native	4		150	0.012556	2.014212
11	nesa	native	5		121.6667	0.012444	2.584548
11	oleu	invasive	1	6.746527	410	0.017333	2.964785
11	oleu	invasive	2	6.988187	388.3333	0.018556	4.199043
11	oleu	invasive	3	7.109017	320	0.018222	4.179678
11	oleu	invasive	4		351.6667	0.017556	3.130965
11	oleu	invasive	5		406.6667	0.018889	3.012914
12	erva	native	1	2.577892	416.6667	0.012556	1.117708
12	erva	native	2	2.698722	415	0.010667	1.125167
12	erva	native	3	3.000797	415	0.011778	0.77625
12	erva	native	4		415	0.010778	0.8113
12	erva	native	5		415	0.012	1.028908
12	paur	invasive	1	4.511172	361.6667	0.009778	0.801724
12	paur	invasive	2	4.571587	381.6667	0.009444	0.814123
12	paur	invasive	3	4.994492	187.5	0.006333	0.602439
12	paur	invasive	4		385	0.010222	0.683699

12	paur	invasive	5		305	0.007667	0.95
13	plha	native	1	3.665362	160	0.02525	1.439583
13	plha	native	2	3.907022	152.5	0.0295	1.281873
13	plha	native	3	4.934077	107.5	0.0215	1.256587
13	plha	native	4		120	0.02475	1.671939
13	plha	native	5		130	0.02225	1.403439
13	plla	invasive	1	6.806942	81.66667	0.011167	1.488935
13	plla	invasive	2	6.686112	95	0.012417	1.613471
13	plla	invasive	3	6.021547	106.6667	0.011667	1.989441
13	plla	invasive	4		76.66667	0.010167	1.842268
13	plla	invasive	5		90	0.01075	1.883347
14	ersa	native	1	5.538227			3.474832
14	ersa	native	2	4.813247			2.069018
14	ersa	native	3	4.571587			3.033831
14	ersa	native	4				2.180821
14	ersa	native	5				
14	prpa	invasive	1	3.484117	240	0.012	4.170913
14	prpa	invasive	2	3.665362	261.6667	0.011333	3.235847
14	prpa	invasive	3		308.3333	0.010667	4.090886
14	prpa	invasive	4		260	0.011	3.50365
14	prpa	invasive	5		198.3333	0.008444	
15	mepo	native	1	14.47965	256.6667	0.016917	1.650755
15	mepo	native	2	15.38587	285	0.01725	1.63729
15	mepo	native	3	17.25874	273.3333	0.019167	1.566941
15	mepo	native	4		305	0.019583	1.671982
15	mepo	native	5		265	0.02025	
15	psca	invasive	1	20.82322	363.3333	0.021333	1.910847
15	psca	invasive	2	19.49409	265	0.019417	1.756167
15	psca	invasive	3	21.36696	250	0.020083	2.145228
15	psca	invasive	4		286.6667	0.02025	1.901903
15	psca	invasive	5		270	0.02075	
16	osan	native	1	13.0901	133.3333	0.010333	1.40633
16	osan	native	2	14.90255	95	0.010583	1.239302
16	osan	native	3	14.17757	158.3333	0.010583	1.093874
16	osan	native	4	13.81508	183.3333	0.009083	1.275125
16	osan	native	5		155	0.01125	0.944457
16	pyan	invasive	1	6.202792	66.66667	0.010583	2.322545
16	pyan	invasive	2	5.840302	96.66667	0.008333	3.074031
16	pyan	invasive	3	7.350677	75	0.010083	2.201814
16	pyan	invasive	4		60	0.01075	2.705938
16	pyan	invasive	5		93.33333	0.011	2.072246

17	heco	native	1	4.934077	405	0.005778	0.655522
17	heco	native	2	5.477812	428.3333	0.006778	0.758732
17	heco	native	3	4.329927	430	0.008556	0.88632
17	heco	native	4		430	0.008222	0.80088
17	heco	native	5		430	0.007444	0.817027
17	rhre	invasive	1	5.054907	385	0.005556	0.786904
17	rhre	invasive	2	4.632002	240	0.005667	0.515585
17	rhre	invasive	3	4.511172	220	0.006944	0.644595
17	rhre	invasive	4	4.511172	181.6667	0.005111	0.616589
17	rhre	invasive	5		193.3333	0.005222	0.786522
18	ruel	invasive	1	12.18388	148.3333	0.006583	1.710193
18	ruel	invasive	2	12.42554	45	0.007833	1.253487
18	ruel	invasive	3	16.35251	35	0.005	1.326393
18	ruel	invasive	4		26.66667	0.007833	1.213455
18	ruel	invasive	5		71.66667	0.007583	1.428353
18	ruha	native	1	11.76097	5	0.004167	0.71134
18	ruha	native	2	13.81508	28.33333	0.005917	0.935885
18	ruha	native	3	11.76097	6.666667	0.003	1.024543
18	ruha	native	4		5	0.002833	1.065448
18	ruha	native	5		16.66667	0.002333	
19	dovi	native	1	10.2506	63.33333	0.014556	1.839973
19	dovi	native	2	9.042297	18.33333	0.013667	2.471134
19	dovi	native	3	8.317317	60	0.012889	1.445529
19	dovi	native	4		51.66667	0.014889	1.533188
19	dovi	native	5		55	0.016333	2.494004
19	scte	invasive	1	12.06305	65	0.013556	2.119737
19	scte	invasive	2	13.21093	75	0.014444	2.30597
19	scte	invasive	3	13.15052	61.66667	0.013222	1.599089
19	scte	invasive	4		48.33333	0.013889	2.048349
19	scte	invasive	5		53.33333	0.013667	1.908082
